# Modified Formula for Designing Ultra-High-Performance Concrete with Experimental Verification

**DOI:** 10.3390/ma13204518

**Published:** 2020-10-12

**Authors:** Jarosław Siwiński, Anna Szcześniak, Adam Stolarski

**Affiliations:** Faculty of Civil Engineering and Geodesy, Military University of Technology, 2 gen. Sylwestra Kaliskiego Street, 00-908 Warsaw, Poland; anna.szczesniak@wat.edu.pl (A.S.); adam.stolarski@wat.edu.pl (A.S.)

**Keywords:** ultra-high-performance concrete, modified design of concrete, experimental verification, fiber reinforcing, mechanical properties, compressive strength

## Abstract

The main purpose of the study was to propose a modification of Larrard’s formula for both the design and compressive-strength evaluation of ultra-high-performance concrete. The proposed modification consisted of the introduction of new parameters into the original formula that allowed it to consider the amount of binders and fine-grained aggregates, the amount of reinforcing fibers, the specimen shape and size, the curing time, and a reinterpretation of the water/cement ratio. The proposed modification was verified based on comparative analysis with the results of our own experimental studies and results taken from the literature. A very good convergence of these results was demonstrated, indicating the validity of the proposed modification.

## 1. Introduction

Ultra-high-performance concrete (UHPC) is defined by the American Concrete Institute (ACI) Committee 239 as “concrete that has a minimum specified compressive strength of 150 MPa (22,000 psi) with specified durability, tensile ductility, and toughness requirements” in which “fibers are generally included to achieve specified requirements” [1]. We can distinguish UHPC concretes with or without steel fiber content.

Reactive powder concretes (RPC) are a specific type of UHPC concrete. Larrard [2] presents the most important information regarding the effect of the blending composition on the technical parameters of the concrete. Many sources provide information on UHPC concretes, e.g., [3,4,5,6], which outline the principles and guidelines for the design and performance of concrete mixes.

The main factors determining the final mechanical performance parameters of UHPC concretes are:

- The W/C ratio within the range of 0.16–0.23, depending on the consistency required.
- The CEM I 52.5 type of cement with a low C3A content and Blaine value of about 4000 cm^2^/g [3].
- The amount of silica fume (SF), with dimensions of 0.1–2.5 µm and C/SF = 1:0. 25 [4].
- The amount of glass powder (GP) or other filler, with dimensions of 0.8–4 µm and similar material parameters, C/GP = 1:0.25 [4].
- The proportions of sand A (80–200 µm) and sand B (400–800 µm) at 30%/70% [4].
- The type of superplasticizer used—polycarboxylate based [7,8], ideally at 0.5%–2.0%.
- A sand-to-cement ratio of 1.1–1.4 [4,9].
- The amount and type of steel fibers. Zemei et al. [10] tested the different proportions of long (13 mm) and short (6 mm) fibers; the best results were obtained for L/S = 1.5%/0.5%. Yang et al. [11], Zemei et al. [12] examined the influence of the type of steel fiber on the strength of the concrete; significant increases in the mechanical parameters of the concrete were achieved by using certain fibers.
- The mixing method, speed and duration. Mazanec et al. [13] presents the calculations for mixing time depending on the tool speed, while Hiremath and Yaragal [14] presented three- and four- stage mixing. The best result was obtained for the B sequence: cement and dry silica fume (1 min, 15–20 RPM), 80% water + 100% SP (3 min, 20–60 RPM), sand and quartz powder (4 min, 20–60 RPM), and 20% water (4 min 90 RPM).
- The type and size of aggregate. Collepardi et al. [15] showed that replacing fine sand with coarse aggregate of maximum size 8 mm had no effect on the compressive strength. Wille et al. [3], reported that ultra-high-performance fiber-reinforced concrete (UHPFRC) including coarse aggregate with a maximum grain size ranging from 7 to 16 mm exhibited a slightly higher compressive strength of 178 MPa, compared to its counterpart without coarse aggregate (162 MPa). In turn, Abdulkareem et al. [8], Siwiński et al. [16], Szcześniak et al. [17], Li et al. [18] present the use of various types of aggregate (basalt, limestone, sand and granite). Yoo and Banthia [19], Rougeau and Borys [20] reported that UHPFRC with SF exhibited the best mechanical properties. Other ultra-fine admixtures (e.g., metakaolin, pulverized FA, limestone micro-filler, siliceous micro-filler, and micronized phonolite) can also be used to achieve compressive strengths above 150 MPa [19,20].

In addition to the many factors mentioned above, the curing conditions are important. The effects of the vacuum mixing and curing conditions are presented, among others, in [21,22,23,24]. The use of curing conditions at elevated temperatures and pressure increases the early strength parameters, mainly by reducing the content of air pores and accelerating the binding reaction. However, it should be noted that the increase in temperature under curing conditions may reduce the strength parameters over a long maturation period, i.e., 90 days.

Considering that so many factors affect concrete’s strength parameters, it is difficult to predict the best values. The basic formula for designing the compressive strength of concrete is Feret’s law, which relates the compressive strength of high-strength concrete (HSC) (of fluid consistency, and having a w/c ratio of less than 0.4) to the water/cement ratio, silica/cement ratio, class of cement and characteristics of the aggregate [25]. A further modification of Feret’s formula includes a coefficient of the effectiveness of the silica fume. Larrard’s formula [25] does not include the amount of dispersed fibers contained in the mixture and the type of aggregate used. The most commonly used model to determine the aggregate composition is the Andreasen and Andersen model modified by Funk and Dinger and described by Yu et al. [26], Shi et al. [27], Sohail et al. [28] along with an optimization algorithm based on the least squares method (LSM). However, to use the algorithm, one must know the exact grain sizes of individual components of the mixture. This requires the use of a scanning electron microscope (SEM), which is not always available to those who design concrete mixes in industry.

In the current literature, the test formulas used do not take into account the type of aggregate, the weight ratio between steel fibers and other aggregate compositions and the strength increase over time. The literature often lacks information about the actual characteristic strength of cement, and only the class is given, which makes it difficult to compare with the results of other authors.

The main purpose of the work was to develop and propose a modification of Larrard’s formula for designing ultra-high-performance concrete. The essence of the proposed modification is the introduction of new parameters to the original formula that allow it to include the amount of binders and fine-grained aggregates, the amount of reinforcing fibers, the specimen shape and size, the curing time, and a reinterpretation of the water/cement ratio. Moreover, the proposed modification was verified on the basis of experimental studies, and a broad comparative analysis was performed with results from the literature. A very good convergence of the results was demonstrated, indicating the validity of the introduced modification.

## 2. Materials and Methods

### 2.1. Materials and Mix Proportions

The cement used in this study was CEM I 52.5 R white with 4725 cm^2^/g Blaine fineness. A polycarboxylic-ether-based superplasticizer (SP) was used to adjust the workability of the UHPC. Two types of microsilica were used: the first (MS1) was a powder with an average particle size (d50) of 0.15 µm, and the second (MS2), with d50 = 65 µm. Both types of microsilica have the same chemical composition because MS2 is formed by the compaction of MS1. One type of the glass powder was selected with d50 = 50 µm. Basalt aggregate with a grain size of 0.125–0.25 mm was used. The chemical compositions of the used powders were determined by X-ray fluorescence (XRF) (Table 1). The uncertainties of the laboratory measurements δ of chemical compounds for individual materials are presented.

The presented materials were used in part of own experimental research, which was carried out for four types of concrete mixes; the mix proportions of these specimens are shown in Table 2. These were developed by following guidelines concerning the C/SF proportions, the Funk and Dinger model for the selection of aggregate component proportions and the modified formula (3) for predicting compressive strength in relation to the amount of water with the assumed amount of cement. Moreover, the results of the preliminary assessment based on the analysis of the literature review and our own previously performed laboratory tests were used.

### 2.2. Methods

In this study, the modified Funk and Dinger model, following the Andreasen and Andersen model, was used in the design of concrete mixes:

(1) $$P(D_{i})=\frac{\left(D_{i}^{n}-D_{\min}^{n}\right)}{\left(D_{\max}^{n}-D_{\min}^{n}\right)}\cdot 100\%$$

where $P\left(D_{i}\right)$ = the cumulative percentage of the i-th fraction lower than $D_{i}$; $D_{i}=\mathrm{the}$ granulation of the calculated fraction (μm); $D_{\min}=$ the granulation of the minimum fraction (μm); $D_{\max}=\mathrm{the}$ granulation of the maximum fraction (μm); n= a constant exponent as the distribution coefficient depending on the type of concrete (composites).

As presented in the literature, different types of concrete can be designed using Equation (1), by applying different values of the constant n, which is determined by the proportions of the fine and coarse particles. In most papers, n = 0.25 is recommended. Here, n = 0.23 was adopted, as it was recommended by [26], considering that a large number of fine particles is used to produce UHPC.

Obtaining high-strength concrete requires the selection of concrete components in proportions and particle sizes ensuring maximum packing density and minimum porosity of the hardened concrete. For this purpose, a modified Funk and Dinger model, well-established in concrete design practice, is commonly used. However, it should be taken into account that the use of very fine cement components and silica fume need not lead to the highest dry packing density due to the electrostatic forces between these components. Detailed information on the model selection and comparison with other modeling methods of mixtures are presented by Sohail et al. [28].

The compressible packing model (CPM) originally developed by Larrard [25] was used as the basis for designing ultra-high-performance concrete:

(2) $$f_{L}=\frac{k_{\mathrm{kL}\;}f_{\mathrm{cem}}\;}{{\left[1+\frac{3.1\;\cdot \frac{W}{C}}{1.4-0.4\cdot \exp\left(-11\cdot \frac{m_{s}}{C}\right)}\right]}^{2}}$$

where $f_{L}=$ the compressive strength after 28 days (MPa); $f_{\mathrm{cem}}=$ the strength of the cement as measured on ISO mortar (MPa); $k_{\mathrm{kL}}=4.91=$ the aggregate coefficient taken as a constant value; $\frac{W}{C}=$ the ratio of water included in all the mixture elements (including water in the SP) to the cement amount; $m_{s}/C$ = the ratio of the microsilica fume to cement.

It should be emphasized that the value of the designed concrete’s compressive strength according to Larrard’s Equation (2) was determined after 28 days for a cylindrical sample with dimensions of 160 mm × 320 mm.

The proposed modification of the formula for designing ultra-high-performance concrete in our study consists of introducing into Equation (2) parameters describing the amount of binders and fine-grained aggregates, the amount of reinforcing fibers, the specimen shape and size, the curing time, and a reinterpretation of the water/cement ratio, in the following form:

(3) $$f_{\mathrm{Lt}}=\frac{k_{k\;}k_{\mathrm{fr}\;}k_{\mathrm{sz}\;}k_{t\;}f_{\mathrm{cem}}\;}{{\left[1+\frac{1.4\;\cdot \frac{W}{C+0.22m_{s}}}{1.4-0.4\cdot \exp\left(-11\cdot \frac{m_{s}}{C}\right)}\right]}^{2}}$$

where $f_{\mathrm{Lt}}=$ the compressive strength at the time of t days (MPa); $k_{k}=3.0+\frac{\rho_{B}-B_{s}}{\rho_{B}}=$ the aggregate coefficient, where $B_{s}$ is the amount of binders and aggregates lower than 0.2 mm in size per kg in 1 m^3^ of the concrete, and $\rho_{B\;}$ is the bulk density of all the aggregates in the sample (kg/m^3^); $k_{\mathrm{fr}}=\exp^{{0.034\rho }_{S}}=$ the reinforcing-fiber coefficient, where $\rho_{S}$ is the percentage ratio of steel-fiber mass to the mass of the cement (%); $k_{\mathrm{sz}}=$ the specimen shape and size coefficient according to Table 3, as recommended by Wille et al. [3];${\;k}_{t}=\left[1-\exp\left(-{\left(\frac{t-0.9}{3}\right)}^{0.6}\right)\right]\;$= the sample-curing-time coefficient as recommended by Graybeal [29], where t is the sample-curing time (days); $\frac{W}{C+0.22m_{s}}=$ the total amount of water included in the all mixture elements (including the water in the SP) relative to the amount of cement with 0.22 microsilica fume.

### 2.3. Curing Conditions, Mixing Procedure and Mechanical Properties

The samples were prepared in 100 × 100 × 100 mm^3^ form for the B1 series and 40 × 40 × 40 mm^3^ form for the B2, B3 and F3 series. A variable-speed planetary mixer was used in the mixing procedure for sample preparation:

- Cement and dry silica fume (5 min);
- A combination of 80% water and 100% SP (5 min, 20–60 RPM);
- Sand and glass powder (1 min) + 20% water (5 min).

After 24 h, samples were disassembled and cured in water at 20 °C in accordance with EN 12390-2:2009 [30]. The mixing was executed under laboratory conditions with dried aggregates and powder materials. The room temperature was maintained at around 20 °C during mixing and testing. After curing for 28 days, the samples were tested for compressive strength, in accordance with the standard EN 12390-3:2009 [31], using a MEGA 6-3000-150 (Form+Test, Riedlingen, Germany) hydraulic press.

## 3. Results and Discussion

### 3.1. Comparison of Experimental Results with Modified Formula (3)

#### 3.1.1. Comparison of the Results of Own Experimental Research

**Figure 1:**
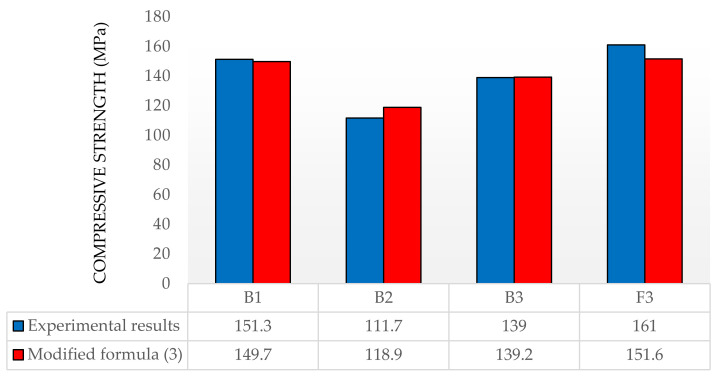
Experimental and designed compressive strength of concrete.

The experimental results for four series (according to Table 2) of specimens with compressive strength designed according to the modified Equation (3) are shown in Figure 1. In order to verify the correctness of the modified formula, high-strength-concrete and UHPC-concrete samples with different aggregate compositions and W/C ratios were produced. MS1 microsilica was used in all the variants. In the B2 series, other than microsilica MS1, compacted microsilica MS2 and crushed quartz GP were used. The use of a large amount of crushed basalt as a fine aggregate resulted in an increase in the W/C ratio, which caused a significant reduction in the compressive strength of the B2 concrete. In the B3 series, MS2 microsilica was not used but quartz-pebble aggregate was used. The F3 series was made with the same composition as the B3 series, but 2% steel fibers were added. The differences in the experimental results and the designed compressive strengths for series B1, B2, B3, and F3 are 1.1%, 9.4%, 0.2%, and 6.2%, respectively (Figure 1). Cone-flow tests were carried out for all the series, and the consistency class was determined as S2 according to the Eurocode standard. As a result of the use of steel fibers, series F3, a 22 MPa increase in the compressive strength of the concrete in relation to the B3 series was obtained (15.8%). The results of the experimental and design studies converged very well.

#### 3.1.2. Comparison of the Results from Experimental Studies by Other Authors

**Figure 2:**
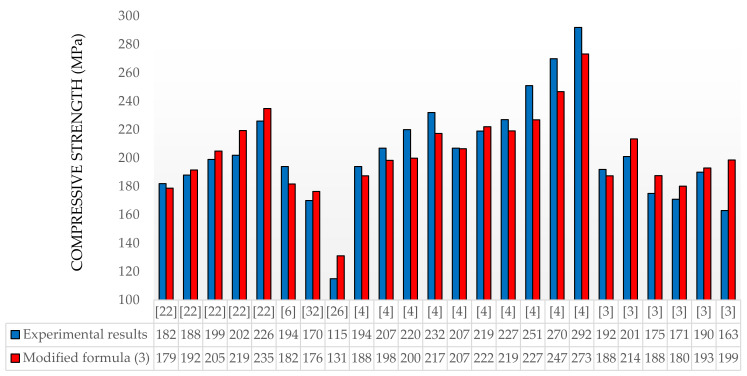
Compressive strength of concrete—experimental results taken from literature [3,4,6,22,26,32] vs. estimations based on the modified formula.

The experimental results of the other authors and compressive strengths designed according to the modified Equation (3) are shown in Figure 2. The mixture proportions are shown in Table 4. In lines 7–10, our own mixture proportions are shown. The average differences in the experimental results in relation to the design results for individual publications are [22]—3.7%; [6]—6.7%; [32]—3.7%; [26]—12.3%; [4]—5.64%; and [3]—4.34% without taking into account the last column in Figure 2 and 6.65% with it for [3]. The difference in the result in the last column is 18%, but this batch should be retested, because UHPC4 [3] differs from the previous UHPC3 [3] recipe in that the water content is 2% lower, while the amount of superplasticizer is 0.18% higher [3].

The observed differences should increase the compressive strength of the concrete. In the case of [26], where the difference in the result is 12.3%, it should be noted that the concrete composition contains aggregate with a maximum aggregate size of 8 mm, cement with a Blaine fineness of 3150 kg/m^3^ and limestone powder filler, which significantly differs from the guidelines presented in the introduction. The use of limestone powder also affects the water supply of the mix composition, which may also affect the final result. It should also be emphasized that the obtained results presented in the paper were determined for the characteristic compressive strength of cement on standard samples. If a standard value is used, e.g., 52.5 MPa, for each of the presented recipes, we obtain correspondingly lower values for the designed compressive strength, which allows for the practical application of the presented formula with a safety margin for concrete. The verification of the formula in relation to the results of other authors showed very good agreement.

### 3.2. Effect of the Amount Fibers on Compressive Strength

**Figure 3:**
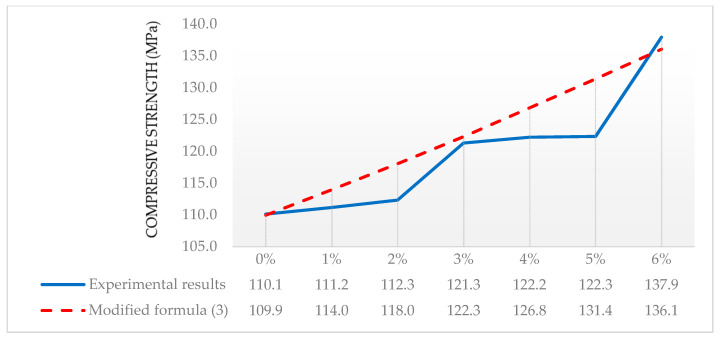
Experimental and designed compressive strength after 28 days for different amounts of fibers [33].

Due to the fact that Pourbaba et al. [33], the characteristic compressive strength of the cement and its class did not give, in order to verify the formula, the concrete class was adopted so that the compressive strength of the concrete samples without fibers was as close as possible. The experimental results of other authors for specimens and the designed compressive strength according to the modified formula, for different amounts of fibers [33], are shown in Figure 3. The mixture proportions are shown in Table 5.

It should be noted that the experimental results obtained for the amounts of steel fibers 1% and 2% differ slightly from the sample made without the addition of dispersed fibers. The difference between Series 0 and 2 is 2.2 MPa (1.96%), which significantly differs from the data—for example, from own research, where a 15.8% increase in compressive strength was obtained, and from the data available in the literature. However, the difference between Series 2 and 3 is already 7.4% and similar to that in [3,4]. Figure 3 shows a flattening of the experimental compressive strength plot between Series 3 and 4 and Series 4 and 5, where the differences are 0.7% and 0.08%, respectively. Another increase in compressive strength is shown in Series 5 and 6, of 11.3%. Despite the doubts regarding the results, it should be noted that a very good average agreement of the results was obtained, 2.6%, with a maximum value of 7.4%.

### 3.3. Comparison of Results for Modified Formula (3) and Larrard Formula against the Background of the Results of the Experimental Research

In order to compare the results obtained with the original Larrard formula (2) with those with the modified formula (3), it is necessary to introduce correction factors to the formula (2) that allow the influence of the shape and size of the samples and the influence of fibers on the compressive strength of the concrete to be determined:

(4) $$f_{\mathrm{LA}}=f_{L}k_{\mathrm{sz}}$$

where $f_{\mathrm{LA}}$ = the compressive strength after 28 days according to the Larrard formula (2) taking into account the coefficient of the sample’s size and shape according to the Table 3 (MPa), and

(5) $$f_{\mathrm{LB}}=f_{L}k_{\mathrm{sz}}k_{\mathrm{fr}}\;$$

where $f_{\mathrm{LB}}$ = the compressive strength after 28 days according to the Larrard formula (2) taking into account the coefficient of the sample’s size and shape and the steel-fiber coefficient according to (3) (MPa).

**Figure 4:**
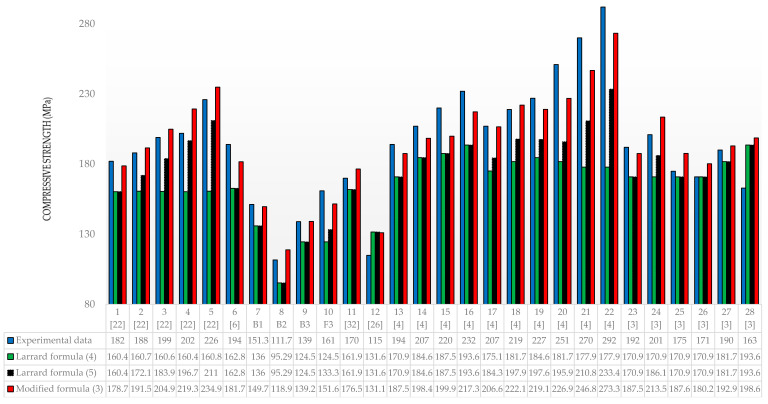
Experimental and design compressive strength for a different mix composition and formulas.

Figure 4 shows the results of our own and other authors’ experimental tests, the results for the designed concrete’s compressive strength according to the modified formula and the results based on the Larrard formula modified according to (4) and (5).

**Figure 5:**
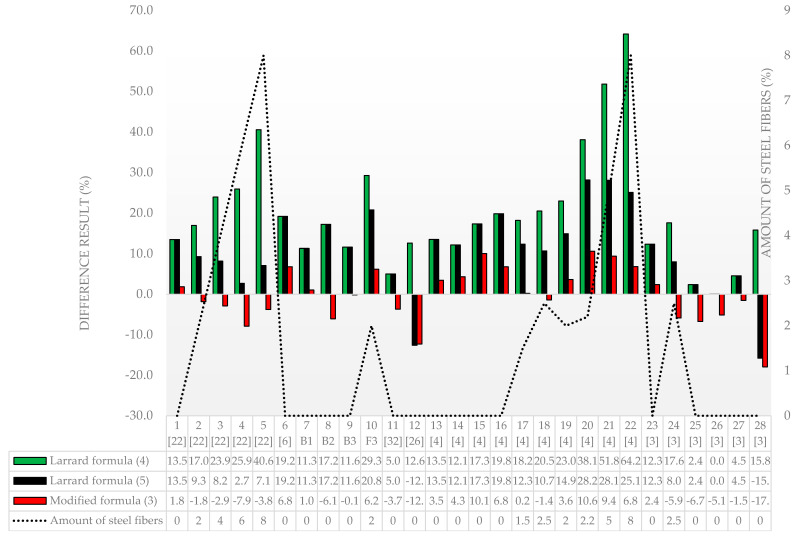
Experimental and design compressive strength for different mix compositions and formulas depending on the amount of steel fibers.

The analysis of the designed compressive strength of concrete (Figure 4 and Figure 5) indicates the following average deviations in relation to the experimental values: Larrard’s formula (4)—19.9%; modified Larrard’s formula (5)—13.0%; the formula modified by the authors—5.4%. The negative values in Figure 5 mean that the results obtained in the experimental tests were lower than the designed result.

The greatest discrepancies in Formula (4) in relation to the experimental compressive strength of concrete were obtained for Columns 22—64.2%; 21—51.8%; and 5—40.6%. It should be noted that these are recipes that add 8%, 5% and 8% steel fibers, respectively, which the basic formula of Larrard does not take into account. In the case of Formula (5), the differences are, respectively, Column 22—25.1%; 21—28.1%; and 5—7.1%. However, for the formula modified by the authors, the differences are Column 22—6.8%; 21—9.4%; and 5—3.8% and show the greatest accuracy in relation to the experimental results. For Formula (8), the greatest discrepancies occur in Columns 20—28.2%; 21—28.1%; and 22—25.1%, for which, using the modified formula, 20—10.6%; 21—9.4%; and 22—25.1% were obtained, respectively.

The average differences in the results for selected papers are shown in Table 6.

We can note that the modified formula obtained the highest accuracy among all the considered publications and the authors’ experimental research.

As a result of comparing 28 different mix designs by many authors, using the formula modified by the authors, the best compliance was obtained for 25 results. For the remaining three results, Columns 4, 25 and 26 in Figure 5, the differences in the final results were insignificant and amounted to 7.9%, 6.7%, and 5.1%, respectively.

## 4. Conclusions

A modified formula for the analytical design of the expected compressive strength of UHPC concretes was proposed. The effect of the amount of steel fibers, the type and amount of aggregate, the grain size, and fillers, as well as the size and shape of the prepared samples, were taken into account. The formulas available in the literature do not take these into account, which significantly affects the experimental results. Thus, the proposed modification of Larrard’s formula enables an objective comparative analysis of the experimental and analytical results obtained for various parameters of UHPC samples. The modified formula can also be used to design high-strength concretes and other cement-based composites.

In many papers concerning the use of Larrard’s formula, the authors do not provide information that this is the compressive strength for a cylindrical sample of 160 mm × 320 mm dimensions. This formula also does not take into account the amount of steel fibers used, which is found in almost every UHPC formula, hence the large discrepancies between the experimental and theoretical results are stated.

The paper presents results of the own experimental tests of four concretes with different compositions, including the use of a combination of steel fibers. The tested concretes were designed using the modified Larrard’s formula. The compressive strength results (151.3; 111.7; 139.0) MPa for samples without fibers and 161.0 MPa for samples with fibers, were obtained. The obtained experimental results differ by those determined on the basis of the modified Larrard’s formula by (+1.0; −6.1; −0.1)% respectively for the samples without fibers and +6.2% for the sample with fibers.

The paper shows a positive verification of the modified Larrard’s formula, obtaining a very good agreement of the calculated results with the experimental results available in the literature.

Using the original Larrard’s formula without taking into account the specimen shape and size coefficient $k_{\mathrm{sz}}$ may lead to discrepancies of up to 11.0%. Taking into account the reinforcing fibers coefficient $k_{\mathrm{fr}}$ reduces the mean error for all recipes in relation to the experimental values, from 19.9% to 13.0%. The verification of the coefficient based on the results presented in [33] shows a very good average agreement between the results was obtained in the range from the minimum value 2.6% to the maximum value of 7.4%, with a mean error value of 5.4%. If we use the formula without taking into account $k_{\mathrm{sz}}$. and $k_{\mathrm{fr}}$, the error in relation to the experimental results can reach 30%.

Nevertheless, Larrard’s equation requires further modifications to adapt to the currently used recipes containing more and more steel fibers. The subsequent modification levels should concern in-depth verification and refine the curing time coefficient $k_{t}$, the specimen shape and size coefficient $k_{\mathrm{sz}}$, the reinforcing fibers coefficient $k_{\mathrm{fr}}$, in which the contribution of other than steel fibers but also mixed types of fibers will be taken into account. Further modifications should be made to take into account the influence of other factors, such as the quality of cement and aggregates, the level of moisture in the components, the technique of producing the mixture, and the maturing methods used.

## Figures and Tables

**Table 1:** Chemical compositions of cement (C), microsilica (MS1 and MS2) and glass powder (GP).

Chemical Composition	C (mass %)	±δC (mass %)	MS1 (mass %)	±δMS1 (mass %)	MS2 (mass %)	±δMS2 (mass %)	GP (mass %)	±δGP (mass %)
CaO	67.42	0.003	0.10	0.067	0.10	0.067	0.38	0.052
SiO_2_	22.56	0.022	96.2	0.012	96.2	0.012	99.70	0.013
Al_2_O_3_	2.69	0.249	-	-	-	-	0.11	0.185
Fe_2_O_3_	0.19	0.067	0.50	0.029	0.50	0.029	0.09	0.033
K_2_O	0.03	0.090	1.30	0.025	1.30	0.025	-	-
SO_3_	2.10	0.037	0.20	0.161	0.20	0.161	0.53	0.125
CaCO_3_	-	-	-	-	-	-	0.3	0.048
MgO	-	-	1.70	0.675	1.70	0.675	1.40	0.583

**Table 2:** Own mixture proportions.

Mix	Cement	MS 1	MS2	GP	Basalt/Sand	Water	SP	Fibers
B1	1	0.22	0	0	1.5 ^1^	0.28	0.033	0
B2	1	0.25	0.3	0.3	0.7 ^2^	0.33	0.035	0
B3	1	0.25	0	0.3	1.0 ^3^	0.25	0.02	0
F3	1	0.25	0	0.3	1.0 ^3^	0.25	0.02	2% ^4^

^1^ basalt, 0.125–0.25 mm, amount 0.57; 0.25–0.5 mm, amount 0.93. ^2^ basalt, 0.125–0.25 mm. ^3^ quartz sand, 0.125–1.0 mm. ^4^ total amount of KrampeHarex steel, brass coated fibers mix of 0.175 mm diameter, 2800 MPa tensile strength, 210 GPa modulus of elasticity: type 1–6 mm length −0.5% and type 2–12 mm length −1.5% .

**Table 3:** Specimen shape and size coefficient $k_{\mathrm{sz}}$ [3].

Specimen Shape	Specimen Size (mm)	$k_{\mathrm{sz}}$
cylinder	150 × 300	1
	100 × 200	1.04
	76 × 150	1.05
cube	150	1.02
	100	1.06
	50	1.10
	40	1.11

**Table 4:** Mixture proportions [3,4,6,22,26,32].

Mix	(Ref.)	Cement	S	MS	GP	SP	Fiber	W
			(<0.2 mm)				In vol. %	
1	[22]	1.00	0.81	0.20	0.35	0.02	0.00	0.24
2	[22]	1.00	0.81	0.20	0.35	0.02	0.00	0.24
3	[22]	1.00	0.81	0.20	0.35	0.02	0.00	0.24
4	[22]	1.00	0.81	0.20	0.35	0.02	0.01	0.24
5	[22]	1.00	0.81	0.20	0.35	0.02	0.01	0.24
6	[6]	1.00	0.81	0.20	0.34	0.02	0.00	0.24
7	B1	1.00	1.50	0.22	0.00	0.03	0.00	0.28
8	B2	1.00	0.70	0.25	0.60	0.04	0.00	0.33
9	B3	1.00	1.00	0.25	0.30	0.02	0.00	0.25
10	F3	1.00	1.00	0.25	0.30	0.02	0.63	0.25
11	[32]	1.00	1.20	0.28	0.30	0.02	0.00	0.24
12	[26]	1.00	1.79	0.37	0.49	0.07	0.00	0.25
13	[4]	1.00	1.10	0.25	0.53	0.01	0.00	0.22
14	[4]	1.00	0.71	0.25	0.55	0.01	0.00	0.20
15	[4]	1.00	0.72	0.25	0.56	0.01	0.00	0.19
16	[4]	1.00	0.00	0.25	1.05	0.01	0.00	0.18
17	[4]	1.00	1.05	0.25	0.52	0.01	1.50	0.21
18	[4]	1.00	0.64	0.25	0.53	0.01	2.50	0.20
19	[4]	1.00	0.67	0.25	0.54	0.01	2.00	0.20
20	[4]	1.00	0.00	0.25	1.17	0.01	2.20	0.20
21	[4]	1.00	0.00	0.25	1.01	0.01	5.00	0.21
22	[4]	1.00	0.00	0.25	0.97	0.01	8.00	0.21
23	[3]	1.00	1.10	0.25	0.53	0.01	0.00	0.22
24	[3]	1.00	1.03	0.25	0.51	0.01	2.50	0.22
25	[3]	1.00	1.18	0.25	0.80	0.01	0.00	0.22
26	[3]	1.00	1.68	0.25	0.30	0.01	0.00	0.22
27	[3]	1.00	1.10	0.25	0.53	0.01	0.00	0.20
28	[3]	1.00	1.09	0.25	052	0.01	0.00	0.18

**Table 5:** Mixture proportions [33].

Mix	(Ref.)	Cement	S	MS	GP	SP	Fiber	W
			(<0.2 mm)				In vol. %	
1	[33]	1.00	1.43	0.33	0.30	0.04	0.00	0.26
2	[33]	1.00	1.43	0.33	0.30	0.04	1.00	0.26
3	[33]	1.00	1.43	0.33	0.30	0.04	2.00	0.26
4	[33]	1.00	1.42	0.33	0.30	0.04	3.00	0.26
5	[33]	1.00	1.42	0.33	0.30	0.04	4.00	0.26
6	[33]	1.00	1.42	0.33	0.30	0.04	5.00	0.26
7	[33]	1.00	1.42	0.33	0.30	0.04	6.00	0.26

**Table 6:** Average differences in the results for [3,4,22] and our own experimental data.

Formula	Paper [22]	Own Exp. Data	Paper [4]	Paper [3]
Larrard (4)	24.2%	17.3%	27.9%	8.8%
Larrard (5)	8.1%	15.2%	18.2%	7.2%
Modified Formula (3)	2.9%	3.4%	5.7%	6.6%
